# Impaired Verbal Learning Is Associated with Larger Caudate Volumes in Early Onset Schizophrenia Spectrum Disorders

**DOI:** 10.1371/journal.pone.0130435

**Published:** 2015-07-31

**Authors:** Monica Juuhl-Langseth, Cecilie B. Hartberg, Aina Holmén, Rune Thormodsen, Inge R. Groote, Lars M. Rimol, Kyrre E. Emblem, Ingrid Agartz, Bjørn R. Rund

**Affiliations:** 1 Research Unit Child and Adolescent Mental Health, Oslo University Hospital, Oslo Norway; 2 NORMENT and K.G. Jebsen Centre for Psychosis Research, Institute of Clinical Medicine, University of Oslo, Oslo, Norway; 3 Department of Psychology, University of Oslo, Oslo, Norway; 4 Mental Health Services, Akershus University Hospital, Lørenskog, Norway; 5 Vestre Viken Hospital Trust, Drammen, Norway; 6 Department of Laboratory Medicine, Children's and Women's Health, Norwegian University of Science and Technology, Trondheim, Norway; 7 The Intervention Centre, Oslo University Hospital, Oslo, Norway; 8 Department of Psychiatric Research, Diakonhjemmet Hospital, Oslo, Norway; Bellvitge Biomedical Research Institute-IDIBELL, SPAIN

## Abstract

**Background:**

Both brain structural abnormalities and neurocognitive impairments are core features of schizophrenia. We have previously reported enlargements in subcortical brain structure volumes and impairment of neurocognitive functioning as measured by the MATRICS Cognitive Consensus Battery (MCCB) in early onset schizophrenia spectrum disorders (EOS). To our knowledge, no previous study has investigated whether neurocognitive performance and volumetric abnormalities in subcortical brain structures are related in EOS.

**Methods:**

Twenty-four patients with EOS and 33 healthy controls (HC) were included in the study. Relationships between the caudate nucleus, the lateral and fourth ventricles volumes and neurocognitive performance were investigated with multivariate linear regression analyses. Intracranial volume, age, antipsychotic medication and IQ were included as independent predictor-variables.

**Results:**

The caudate volume was negatively correlated with verbal learning performance uniquely in the EOS group (r=-.454, p=.034). There were comparable positive correlations between the lateral ventricular volume and the processing speed, attention and reasoning and problem solving domains for both the EOS patients and the healthy controls. Antipsychotic medication was related to ventricular enlargements, but did not affect the brain structure-function relationship.

**Conclusion:**

Enlargement of the caudate volume was related to poorer verbal learning performance in patients with EOS. Despite a 32% enlargement of the lateral ventricles in the EOS group, associations to processing speed, attention and reasoning and problem solving were similar for both the EOS and the HC groups.

## Introduction

Schizophrenia is a disorder of the brain in which structural abnormalities and impairment of neurocognitive function are features that may reflect underlying neurodevelopmental pathology [1–4]. Neurocognitive functioning is usually considered in relation to neocortical structure and function, but subcortical brain structures are also implicated in cognitive processing through multiple cortico-striatal loops [5–9]. Studies of patients with adult onset schizophrenia have reported relationships between the size of specific subcortical brain structure volumes and the severity of cognitive dysfunctions [10–13].

Approximately 5% of schizophrenia patients have EOS, i.e. illness onset before the age of 18 [14]. Current evidence shows a continuity between EOS and adult onset schizophrenia, with phenomenological, cognitive, genetic and neuroimaging data pointing toward similar neurobiological correlates and clinical deficits [15, 16]. To date, most studies conclude that EOS constitutes a more severe presentation of the disorder, with a worse long-term outcome [17, 18]. More optimistically, recent reports conclude that better symptomatic and functional outcomes were recorded for EOS patients compared to adult onset schizophrenia when detected early and given specialized treatment [19].

Both brain structural and neurocognitive abnormalities are reported in EOS [20–25]. In a previous study, we investigated 27 subcortical brain structure volumes in patients in the narrow EOS spectrum (not including psychotic disorder NOS), and reported an enlargement of the caudate nucleus, the lateral and the fourth ventricles volumes compared to the healthy control (HC) group [22]. Antipsychotic medication was related to lateral ventricle enlargements, and most likely the bilateral enlargements of caudate volumes. Furthermore, impairment in the six neurocognitive domains of the MATRICS Cognitive Consensus Battery (MCCB)[26, 27] has previously been reported [23, 28].

Investigating the relationship between brain structure volume and neurocognitive function may provide insights into the neurodevelopmental underpinnings of schizophrenia [29]. In a review of adult onset schizophrenia, it was concluded that there is strong evidence to suggest that progressive changes occur early in the course of the illness [30]. In this context, studies of patients with EOS provide an opportunity to explore putative brain pathology free from the potential confounding effects of long-term medication, age-related neurodegeneration and several of the suggested disease risk-related environmental factors [31, 32].

The caudate nucleus is part of the basal ganglia, which receive widespread input from the cerebral cortex and the thalamus, and projections are returned through output in thalamocortical pathways [33] and contribute to a wide range of cognitive functions, including learning, memory, executive functions and the processing of rewards and other feedback [9, 34]. The basal ganglia are characterized by prominent dopaminergic pathways and represent major locations of synaptic plasticity [35–37]. The dopamine hypothesis has been central in schizophrenia research since the 1950s, and contemporary pathophysiological models assume that psychosis is triggered by dysregulation of dopaminergic activity in the brain [38, 39].

The ventricular system contains cerebrospinal fluid that has a number of important functions, including cushioning and protection of the brain, removal of waste material and transport of hormones and other substances [40]. The most consistently reported brain structure abnormality in schizophrenia is enlarged ventricles, which is found in 80% of the studies in adult onset schizophrenia [2, 41]. However, the role of the increased ventricular system in schizophrenia is still largely unknown. Substantial evidence relates both the caudate and the lateral ventricles enlargements to exposure to antipsychotic medication, including both first and second generation compounds [22, 42–45]; however, some studies have not found relationships for second-generation antipsychotics [46].

The aim of the current study was to test whether there was a relationship between neurocognitive performance measured by the MCCB and the brain structure volumes (caudate nucleus and the lateral and fourth ventricles) found to be enlarged in our previous study of the same EOS patients [22]. Putative effects of antipsychotic medication to any relationships between brain structure and cognition were investigated.

## Materials and Methods

### Subjects

The patients were participants in a broader research project on early onset psychosis conducted at the University of Oslo, Norway [24, 25, 28]. Inclusion criteria were age of illness onset below 18 years a diagnosis within the broader schizophrenia spectrum (schizophrenia or schizoaffective disorder or psychotic disorder not otherwise specified (NOS)), in order represents the larger schizophrenia spectrum disorders. This is a difference from the patient population in our previous study where patients with psychosis NOS (n = 6) were excluded [22]. In the current study, 24 out of a total of 28 scanned patients were used for further analyses. F our subjects were excluded due to poor image quality related to movement or dental braces in four subjects. The HC group consisted of 33 research participants recruited through letters to a group of randomly selected individuals from the Norwegian population, as well as through advertisements in four schools in the greater Oslo area. All the controls attended regular school at normal grade levels, and the group was screened for mental problems using The Mini-International Neuropsychiatric Interview (M.I.N.I.) screening module [47]. A positive response to any of the questions was grounds for exclusion from the study. Clinical and demographic data are presented in Table 1.

**Table 1:** Clinical and demographic data for the EOS patients and HC.

	EOS (*n* = 24) Mean (SD)	HC (*n* = 33) Mean (SD)	Test statistics
Number of females (%)	12 (50)	17 (52)	n.s.
Right hand dominance (%)	20 (83)	30 (91)	n.s.
Age at scan (years)	16.1 (1.9)	15.8 (1.8)	n.s.
Mother’s education (years)^a^	13.6 (2.6)	15.5 (2.8)	t(54) = -2.484, **p = .016** *
Father’s education (years)^b^	14.1 (2.8)	14.4. (3.1)	n.s.
Full-scale IQ (WASI)	96.8 (16.3)	107.3 (14.6)	t(55) = -2.524, **p = .015** *
Schizophrenia (n/%)	15 (62.5)		
Schizoaffective (n/%)	3 (12.5)		
Psychosis NOS (n/%)	6 (25)		
Age at onset of disorder (years)	14.5 (2.0)		
Duration of illness (years)	1.5 (1.0)		
GAF—function	47.8 (15.4)		
GAF—symptom	49.9 (14.0)		
PANSS—positive	14.3 (4.3)		
PANSS—negative	12.3 (5.7)		
PANSS—general	29.7 (7.5)		
PANSS—total	57.3 (13.4)		
DDD (n = 17/71%)	1.1 (0.7)		
Duration of antipsychotic medication (days) n = 16	168.6 (160) range 19–707		
DUP (weeks)	33 (44.6) range 1–208		

^a^ n = 22 for the patient group

^b^ n = 21 for the patient group

* p<.05

General exclusion criteria were any known brain injury or neurological disease, along with standard contraindications for MRI (metal in the body or cardiac pacemaker) and an IQ<70. A clinical neuroradiology specialist evaluated all of the image series for organic brain pathology.

Written informed consent was obtained from both patients and controls, as well as from their parents if the adolescent was younger than 16 years of age. According to the Norwegian Health Laws, minors between 16 to 18 years of age can consult and consent to medical treatment independently of parental approval. The capacity for the patients to consent to the study was determined by their primary physicians, their parents and by our project group. Both consent forms and procedures for the study were approved by the local Regional Committee for Medical Research Ethics and the Norwegian Data Inspectorate.

### Clinical assessment

As described previously [28, 48], diagnostics were carried out using the Structural Clinical Instrument of Diagnosis for DSM-IV Axis I disorders (SCID-I), modules A-D. All of the interviewers were clinical psychologists who had participated in a training course in SCID assessment based on a training program for this purpose. The mean overall *kappa* for the SCID was 0.77. Psychiatric symptoms and level of functioning were assessed using the Positive and Negative Syndrome Scale (PANSS)[49] and the Global Assessment of Functioning–Split Version (Split-GAF)[50]. An estimated duration of untreated psychosis (DUP) was defined as the number of weeks between the first time any positive symptom on the PANSS reached four points and the first time the patients received psychiatric treatment (with or without antipsychotic medication) such as psycho-education and cognitive therapy. To help obtain a reliable measure of DUP, information from interviews with the patients, parents, treating clinicians and journal data were collected and thoroughly discussed within the research group (AH, MJL and RT) for each patient. Table 1 summarizes the clinical and demographic data in both the patient and HC groups.

### Neurocognitive assessments

Clinical psychologists trained in neurocognitive testing assessed all research participants using the complete MCCB battery. The present study included the following domains [26]: 1) Speed of processing, 2) Attention, 3) Working Memory, 4) Verbal Learning, 5) Visual Learning, and 6) Reasoning and Problem Solving. The seventh domain, Social Cognition, was excluded since we previously found that this test was not sensitive to social cognitive impairments within this group [23]. Four missing test results were replaced by the mean value on the specific test for the EOS group, and the calculation of the IQ estimate was based on the four sub-tests of the Wechsler Abbreviated Scale of Intelligence (Vocabulary, Similarities, Block Design and Matrix Reasoning)[51].

### Antipsychotic medication

Seventeen patients (71%) received antipsychotic medication, while seven patients did not receive any antipsychotic medication. All of the medicated patients received second-generation antipsychotic medication (Aripiprazole, Olanzapine, Quetiapine, Risperidone or Ziprazidone), while two patients (8%) received a combination of first- (Chlorprothixene or Haloperidol) and second-generation antipsychotic medication. For the purpose of statistical analysis, the dosages were converted to defined daily dose (DDD) (http://www.whocc.no/ddd/definition_and_general_considera/). The duration of antipsychotic medication is reported in days given in Table 1.

### MRI acquisition

All images were acquired using a 1.5 Tesla Siemens Sonata scanner (Siemens Medical Systems, Erlangen, Germany), and also included two high-resolution 3D Spoiled Gradient Recalled (3D-SPGR) T1-weighted image series, with the following acquisition parameters applied: 124 contiguous 1 mm sagittal slices, flip angle = 35°, repetition time (TR) = 24 ms, echo time (TE) = 6.0 ms, field of view (FOV) = 256 mm, acquisition matrix = 256 × 256, yielding an isotropic voxel size of 1 mm^3^. In addition, a whole-brain, dual-echo (for varying image contrast) coronal T2 turbo spin-echo sequence (TR = 8000 ms, TE1 = 11 ms, TE2 = 89 ms), 128 slices, slice thickness 2 mm, FOV = 256 mm, acquisition matrix = 256 × 214, was acquired for each patient. All patients and controls were examined during the same study period, and there was no scanner upgrade during this time.

### Image segmentation

All image segmentation was performed using the automatic brain segmentation software tool FreeSurfer version 4.0.4. The two T1-weighted images were averaged and rigid-body registered to each other in order to increase the signal-to-noise ratio. Subcortical volumes were estimated from the fully automated procedure for volumetric estimations of subcortical brain structures implemented in FreeSurfer, which has previously been shown to be comparable in accuracy to manual labeling [52]. Based on our previous findings, the caudate nucleus, the lateral ventricle and the fourth ventricle volumes were selected for the study.

Measurements of ICV were performed with a semi-automated procedure using the software tool Brain Voyager QX (Brain Innovation, Maastricht, the Netherlands). The dual-echo PD/T2-weighted image series was iso-voxelated and co-registered to the T1-weighted image series using sinc interpolation, so that all image series were accessible as overlays for the correct determination of the ICV. Semi-automated segmentation was performed by primarily using the proton density-weighted image series. The T1/T2-weighted image series was consulted whenever there was doubt as to which tissue class a voxel belonged to, and in order to measure reliability, five randomly selected subjects were chosen for the re-measuring of their ICV volumes. The same rater performed this procedure, and the intra-rater reliability correlation was 0.99.

### Statistical analyses

Data analyses were performed with IBM SPSS Statistics, version 20. For the demographics, two-tailed students *t*-tests were used for group comparisons of continuous variables and chi-squares for the group comparisons of categorical data.

Both brain volumes and MCCB domain scores were normally distributed. The brain structures volumes were investigated for covariance using partial correlation analysis.

The brain volumes were investigated for hemispheric laterality for both volumes and structure-function relationships. Because there were no differences between left and right side volumes and volume-function relationship, right and left side were collated in order to reduce the number of statistical tests.

Group differences in performance on the MCCB domains were investigated using two-way t-tests and the raw scores. Group differences for total lateral ventricular, fourth ventricle and caudate nucleus were analyzed using analysis of covariance (ANCOVA) with ICV and age as covariates.

The results were considered significant at p<0.05.

#### Brain structure—neurocognitive function relationships

In order to investigate associations between the brain structure volumes and the MCCB performance, we performed six multivariate linear regression analyses (separated for patients and controls) with each of the structure volumes as the dependent variables and the neuropsychological scores, plus the possible confounders (ICV, age at scan, IQ and antipsychotic medication) as independent-predictor variables. For any significant structure-function correlations, Fisher’s r-to-z transformations were computed. To determine whether there were between-group differences in the correlation coefficients, the z-scores were tested using t-tests (at http://vassarstats.net/rdiff.html).

Relationships between brain structure volumes and MCCB scores with psychiatric symptom and function ratings (the PANSS subscores and the split-GAF scores) as well as DUP, total duration of antipsychotic medication (total number of days) and DDD were investigated for all patients (added as independent predictor-variables) in separate linear regression analyses with each of the brain structure volumes as dependent variables.

In order to investigate the potential influence of diagnosis, the patient group was divided into one schizophrenia group (schizophrenia and schizoaffective disorders; n = 18) and a psychosis NOS group (n = 6). The brain volumes, the MCCB domains and any relevant brain volume/MCCB domain relationship were compared between the two patient groups.

All test results were considered significant at p <.05.

## Results

### Clinical and demographic variables

Table 1 presents demographic and clinical variables. The HC had a significantly higher current full-scale IQ and more years of mothers’ education than the patients. There were no group differences in age, gender, fathers’ education or handedness.

### Brain structure volumes and the MCCB domains

Table 2 presents the results for the group comparisons of the brain structure volumes and the MCCB domains, respectively. There were significant group differences on all measures.

**Table 2:** Group comparisons of the brain structure volumes and the MCCB domains.

	**EOS**		**HC**			
**Brain Volume**	**Estimated Marginal Means**	**Standard Error**	**Estimated Marginal Means**	**Standard Error**	**F**	**p**
Caudate	8,421	148	7,799	127	10.57	**.002** **
Lateral ventricles	14,738	1,140	11,151	971	5.718	**.020** *
Fourth ventricle	1,986	92	1,726	78	4.600	**.037** *
**Neurocognitive Domain**	**Mean (raw scores)**	**S.D.**	**Mean (raw scores)**	**S.D**	**t (df = 55)**	**P**
Processing Speed	40.5	8.1	49.1	8.6	-3.816	**.000** **
Attention	1.7	0.6	2.2	0.6	-2.664	**.010** **
Working Memory	13.3	2.4	17.2	2.9	-5.408	**.000** **
Verbal Learning	23.3	5.7	29.2	3.6	-4.848	**.000** **
Visual Learning	24.4	8.1	28.6	4.3	-2.503	**.015** *
Reasoning and Problem Solving	17.8	5.6	21.9	4.5	-3.062	**.003** **

* p<.05

** p<.01

There were trend-level negative correlations between the PANSS total score, and the Verbal Learning domain (r = -.421, p = .051) and the Processing Speed domain (r = -.402, p = .064).

No significant differences were found in any of the three brain structure volumes between the patients with a diagnosis of schizophrenia or schizoaffective (n = 18) versus psychosis NOS (n = 6) using ANCOVA.

### Associations between the brain structure volumes and the MCCB domains

Table 3 presents the correlation coefficients between each of the three brain structure volumes and the MCCB domain scores, after corrections for possible confounders.

**Table 3:** Linear regression relationships between brain structure volumes and the MCCB domain scores.

		Lateral Ventricles		Fourth Ventricle		Caudate		Fisher’s z-test
		EOS	HC	EOS	HC	EOS	HC	
**Processing Speed**	*Correlation*	**.451**	**.487**	-.145	.103	.179	.161	n.s.
	*Significance*	**.014** *	**.002** *	.249	.284	.201	.186	
**Attention**	*Correlation*	**.530**	**.480**	.041	.147	.294	-.002	
	*Significance*	**.004** *	**.002** *	.424	.207	.082	.495	n.s.
**Working Memory**	*Correlation*	.317	.327	.128	.121	.017	.082	
	*Significance*	.066	.032	.275	.251	.468	.325	
**Verbal Learning**	*Correlation*	-.132	.300	-.179	.160	**-.462**	.149	z = -2.28
	*Significance*	.269	.045	.201	.186	**.012** *	.203	**.023** *
**Visual Learning**	*Correlation*	.192	-.004	.266	.038	.138	-.112	
	*Significance*	.184	.491	.105	.416	.260	.268	
**Reasoning and Problem Solving**	*Correlation*	**.413**	**.367**	.064	.254	.057	**.342**	n.s.
	*Significance*	**.023** *	**.018** *	.383	.077	.396	**.026** *	

* p<.05

There were no structure-function differences between the more narrow schizophrenia subgroup (n = 18) and the patients with psychosis NOS (n = 6).

The structure-function correlations remained unchanged when the PANSS subscale scores and the split-GAF scores were added to the group of confounders in separate analyses for each brain structure volume. There was a significant relationship between the GAF symptom measure and the volume of the lateral ventricles (r = -.437, p = .029).

**Fig 1:**
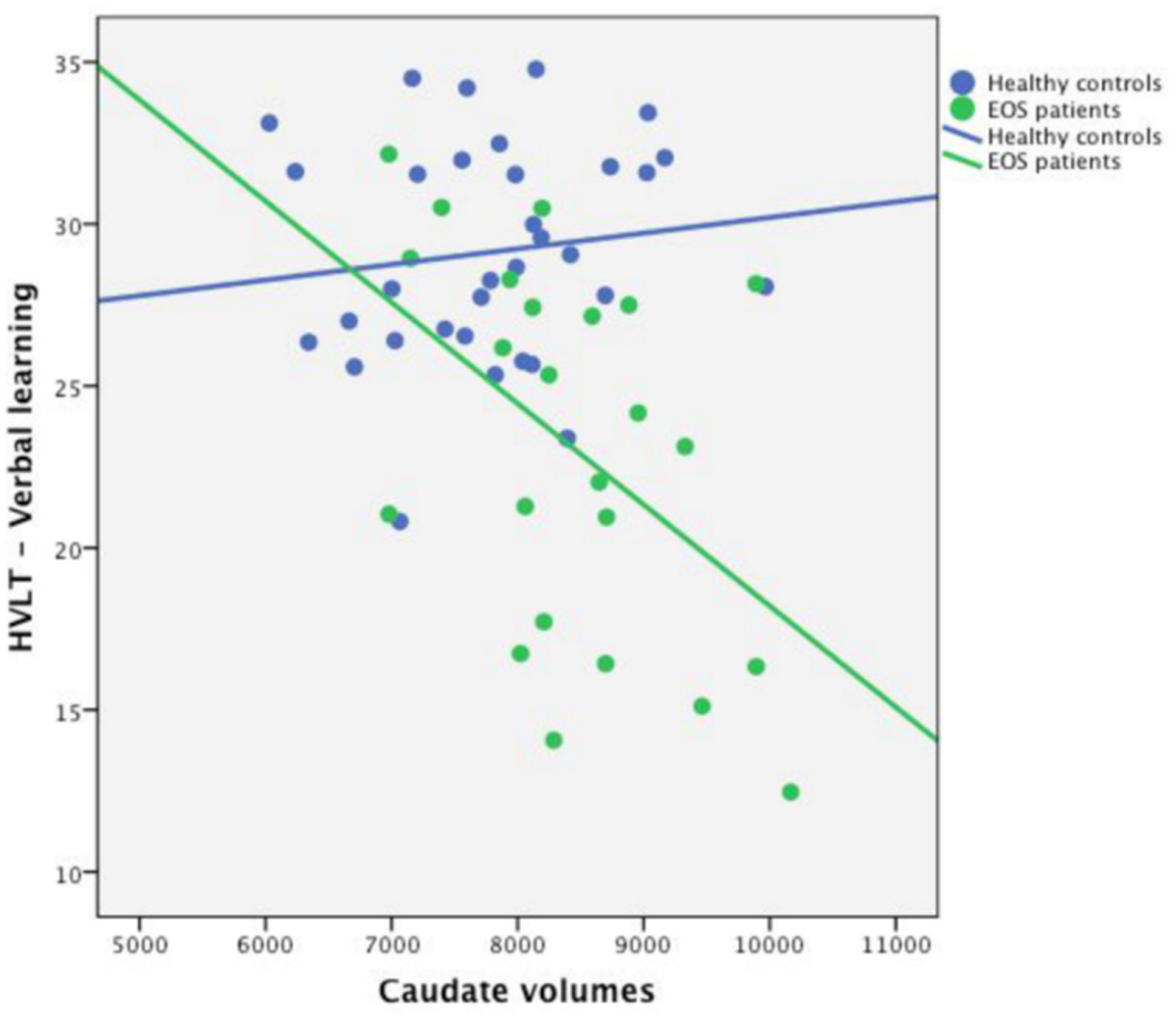
Relationship between the caudate volume and the verbal learning domain in EOS and the HC.

Fig 1 presents the relationships between the caudate volume and the verbal learning domain in EOS and the HC.

### Relationships to medication and symptom scores

There was a significant positive correlation between the lateral ventricle volume and DDD of antipsychotic medication (r = .366, p = .039). There were no relationships to duration of antipsychotic treatment or DUP for any brain structure volume.

There were no statistical relationships between the PANSS and Split-GAF scores and the brain structure volumes, with the exception of a negative correlation between the lateral ventricle volume and the GAF symptom score (r = -.437, p = .029), i.e.. larger ventricle volumes related to more symptoms.

### Additional analyses

Despite increases in both the caudate (5% increase) and the lateral ventricle (25% increase) volumes in medicated patients compared to non-medicated patients, the group differences were not statistically significant.

There were no statistical group differences between medicated and non-medicated patients on the Verbal Learning domain (mean score 22.9 (SD = 5.9) versus 24.1 (SD = 5.6)) for the unmedicated patients.

The correlation coefficients for the caudate volume and verbal learning relationships were not statistically different between the medicated and the non-medicated patient groups.

## Discussion

The main finding in this study was a negative correlation between the caudate volume and the verbal learning performance in the EOS group, i.e. larger caudate volumes were related to poorer verbal learning performance. This relationship was significantly different from that in the HC. A second finding was that correlations between the lateral ventricular volume and processing speed, attention and reasoning and problem solving were similar in EOS and HC. There was also a significant correlation between the caudate volume and the reasoning and problem solving domain for the HC, however the relationship between these measures was not statistically different from the relationship between these measures for the EOS group.

The findings show relationships between cognitive functions and the caudate volume. However, most of the neurocognitive measures were unrelated to the caudate volume. This could mean that the enlargement as such does not predict neurocognitive deficits in EOS, or it may also indicate that total caudate volume is not a sensitive measure for an investigation of relationships to neurocognitive functioning. However, two studies of healthy subjects have reported that caudate volume is related to neurocognitive function. For boys aged 4–18, a positive correlation between IQ and left striatal volumes (adjusted for total brain volume) has been reported [53]. The second study found a negative correlation between gray matter density in the caudate and full-scale IQ regardless of gender [54]. In accordance with these findings, the present study demonstrates a positive relationship between the caudate volume and the reasoning and problem solving domain for the HC group. Taken together, the results indicate that caudate volume is important for normal cognitive functioning. In the current study there was a positive relationship between the caudate volume and the reasoning and problem solving domain for the HC group, indicating that the volume of this structure may relate to cognitive processing efficiency for executive functions. While studies of lesions and direct electrical stimulation of the head of the caudate have found this structure to be involved in various language functions and semantic processing [55, 56], a recent study on the neural basis of language processing suggests that it is probable that direct electrical stimulation interrupts a more general striato-cortical executive “amodal” loop controlling language and other cognitive functions [57].

There was a non-significant trend relationship between verbal learning and PANSS total scores, suggesting a relationship between illness severity and verbal impairment. Verbal learning was the most severely affected domain in our baseline study on an overlapping subject sample, with a mean standard deviation of -1.8 compared to the HC [58]. Interestingly, verbal learning and memory have been found to be the most consistently and significantly affected domains in EOS [21]. However, symptom assessment intertwines with antipsychotic medication, as the more severely ill patients may receive more medication, which in turn can result in lower symptom scores. Therefore, analysis of the relationships between antipsychotic medication effects, brain structure, neurocognitive functioning and psychiatric symptoms is complicated and unique effects are difficult to investigate. Antipsychotic medication is effective for the treatment of positive symptoms in EOS, but has only a small effect on negative symptoms and neurocognitive functioning [59, 60].

In contrast to a significant amount of research and findings for the understanding of neurodevelopmental trajectories in cortical brain volumes [61], few studies have investigated relationships between subcortical brain structure volumes and neurocognitive function in schizophrenia. To the best of our knowledge, there is no such study in EOS. Some studies of adult schizophrenia have failed to find any relationship between subcortical brain structure measures and neurocognitive functioning [62, 63]. However, others have found relationships between putamen and verbal learning, executive and working memory performance [64], between caudate and putamen volumes, with attention and vigilance [65] and between caudate and working memory [66].

More recent studies indicate that shape and not size is a more sensitive measure of the association between neuroanatomical brain structures and neurocognitive performance. In a study of preadolescent children, an expanded shape of the putamen and caudate nucleus was related to poorer performance on tests of neurocognitive function, but not with regard to volume [67]. Basal ganglia regional shapes also correlate with fluid intelligence (abstract reasoning and rule extraction abilities) and spatial intelligence (the spatial manipulation of working memory) of the right striatal structures (the caudate, putamen and nucleus accumbens), but not crystallized intelligence (verbal and numerical reasoning) in adults [68]. In a study of shape measures (local width and local deformation) of the caudate and the hippocampus in patients with adult onset schizophrenia, there were shape deformities in patients compared to the HC [69]. The study had a longitudinal design, and reported no shape changes between 3 to 12 months of treatment with antipsychotic medications.

Another methodological refinement could be to distinguish between, e.g. the ventral-and dorsal caudate regions, involved in learning and response executions respectively [68]. A recent meta-analytic review of fMRI studies presented a comprehensive “topographical map” of how body and eye movements, cognitive functions, emotional and reward processes and somatosensory functions are been regionally represented within the basal ganglia [8], thereby suggesting that a sub-segmentation of these structures may contribute to more refined analyses of structure-function relationships.

Interestingly, despite a 32% ventricular volume enlargement *and* a relationship between DDD of antipsychotics and the lateral ventricles in the EOS group, which suggest that medication confounds structure-function relationships, there were equal relationships for the processing speed, attention and reasoning and problem solving domains for the EOS and the HC groups, i.e. larger ventricular volumes related to better neurocognitive functioning. A review of brain structure and cognition in schizophrenia has also concluded that the relationship between ventricular size and cognitive function is complex [11]. While an enlargement of the ventricles has been found to be related to reduced motor speed in one study of adult onset schizophrenia [10], the opposite finding has also been reported, with larger ventricular size was associated with a higher IQ and better cognitive functioning [70].

In a previous publication, we argued that a positive correlation between antipsychotic medication dosage and ventricular volume could be attributed to more severe underlying brain abnormalities and a more serious case of disease reflected in larger cerebral ventricles [22]. The argument is supported by the finding of a significant relationship between the GAF symptom score and the lateral ventricular volume. However, with regard to the present findings of *better* neurocognitive functioning in patients with larger ventricular volumes, this argument seems weakened, unless better neurocognitive functioning is related to more antipsychotic medication.

In the hitherto longest follow-up study of adult schizophrenia, Andreasen et al. found antipsychotic treatment intensity to be related to brain volume changes, and suggested that clinicians should strive to use the lowest possible dosage to control symptoms [45]. In this context, it is of clinical interest that in the present cross-sectional study, we find that enlarged lateral ventricles showed a similar association to processing speed, attention and reasoning and problem solving for both patients and controls. These findings could indicate that neurocognition and brain structure development follow separate trajectories [28, 71–73]. The enlarged caudate-reduced verbal learning relationship may suggest that antipsychotic medication affect some aspect of the verbal learning neurocognitive process. With regard to relationship between antipsychotic medication and neurocognitive function (including verbal learning), studies report different effects with different neuroleptic agents, and the findings are inconsistent [74]. More generally, most studies have been statistically underpowered to detect moderate effect sizes [60]. Another challenge is that most studies have been on stable chronic patients. Upcoming future studies on patients in the early stages of illness, will possibly elucidate which subjects are most likely to show an effect in neurocognitive function [60]. As the level of neurocognitive impairment best predicts functional outcome in schizophrenia, potential detrimental effects on neurocognitive function are of clinical relevance [75].

### Strengths and limitations

To our knowledge, this is the first study to investigate the subcortical brain structure-neurocognitive function relationships in EOS. The methods and assessment tools used are well validated and recommended for schizophrenia research [76]. The main limitation of the study is the low number of subjects in both groups, and type 2 errors cannot be excluded. Furthermore, since this was a naturalistic history study, we did not have adequate control over antipsychotic medication effects. Relationships between brain structure and neurocognitive function should be investigated both prior to- and after the administration of antipsychotic medication. More studies investigating the relationship between brain structures, antipsychotic- and neurocognitive functioning are needed in EOS.

## Conclusion

For the EOS patients, enlarged caudate volume was related to poorer performance on the verbal learning domain. Interestingly, the associations between the lateral ventricles and processing speed, attention and reasoning and problem solving domains were similar in both EOS and HC despite a 32% ventricular enlargement in the patient group.

## Supporting Information

S1 File Dataset MJL et al 2015 for PLOS ONE. (XLSX)
